# Stigmatization of people who inject drugs (PWID) by pharmacists in Tajikistan: sociocultural context and implications for a pharmacy-based prevention approach

**DOI:** 10.1186/s12954-017-0190-x

**Published:** 2017-09-16

**Authors:** Umedjon Ibragimov, Hannah L. Cooper, Regine Haardörfer, Kristin L. Dunkle, William A. Zule, Frank Y. Wong

**Affiliations:** 1 HIV/AIDS and Harm Reduction Association of Tajikistan, 73/3 I. Somoni Str., office 59, 734064 Dushanbe, Tajikistan; 2 Department of Behavioral Sciences & Health Education, Emory University Rollins School of Public Health, 1518 Clifton Rd NE, Atlanta, GA 30032 USA; 3 Gender and Health Division, South African Medical Research Council, Francie van Zijl Drive, Parowvallei, PO Box 19070, Cape, Tygerberg 7505 South Africa; 4 RTI International, 3040 East Cornwallis Road, P.O. Box 12194, Research Triangle Park, NC 27709 USA; 5 Department of Tropical Medicine, John A. Burns School of Medicine, University of Hawaiʻi at Mānoa, 651 Ilalo St, Honolulu, HI 96813 USA; 6 School of Public Health, Fudan University, 220 Handan Rd, WuJiaoChang, Yangpu Qu, Shanghai Shi, 200433 China

**Keywords:** Pharmacists, Stigma, Injecting drug use, Harm reduction, Tajikistan

## Abstract

**Background:**

Pharmacies are an important source of sterile syringes for people who inject drugs (PWID) in Tajikistan who are under high risk of HIV and hepatitis C virus. Accessibility of sterile syringes at pharmacies without prescription may depend on pharmacists’ attitudes towards PWID. This qualitative inquiry examines meanings and processes of stigmatization of PWID among pharmacists and pharmacy students in Tajikistan.

**Methods:**

We conducted semi-structured interviews with 19 pharmacists and 9 students (*N* = 28) in the cities of Dushanbe and Kulob, Tajikistan. The interview topics included personal attitudes towards drug use and PWID, encounters with PWID, awareness and beliefs related to drug dependence and HIV, and attitudes and practices related to providing syringes to PWID. Interview transcripts were analysed using thematic analysis methods.

**Results:**

The main themes included the significance of religion in defining attitudes towards drug use, labelling of PWID, negative stereotypes (PWID are prone to crime, violence, and irrational aggression; inflict harm to families and society; are able to control drug use), emotions triggered by PWID (fear, sympathy) and discrimination against PWID (rejection, isolation, ostracism, limiting resources to PWID). The religious ban on drug use and pharmacists’ moral and legal responsibility for the consequences of drug use were frequently mentioned as reasons for rejecting syringe sales. Still, many participants acknowledged the need for distributing syringes to PWID to prevent HIV.

**Conclusions:**

Stigma against PWID in Tajikistan plays an important role in shaping pharmacists’ attitudes towards provision of services to this population. Local sociocultural context, in particular religious beliefs and social conservatism, may facilitate stigmatizing beliefs.

## Background

Tajikistan, with a population of 8 million, is one of the poorest countries in post-Soviet Central Asia [1]. Similar to other countries in the region, Tajikistan is experiencing twin epidemics of HIV and hepatitis C virus (HCV) among people who inject drugs (PWID). According to the most recent estimates, HIV and HCV prevalence among PWID (estimated population size = 23,500) in Tajikistan reached 12.9 and 22.7% respectively in 2014 [2, 3]. While about 88% of surveyed PWID reported using sterile injecting equipment during the last injection in 2014, almost a quarter of new HIV cases in the country were still attributed to sharing injecting equipment [2]. If unaddressed, this situation may increase the burden of blood-borne infections among PWID and cause the spread of the HIV epidemic to the general population, as demonstrated in other developing countries [4–6].

Providing access to sterile needles and syringes has been shown to reduce the prevalence of risky injecting practices, and thus contributes to curbing HIV and HCV epidemics among PWID [7–9]. While community-based outreach needle and syringe programmes (NSPs) operate in Tajikistan since 2001, they are not able to meet fully demand of PWID for sterile injecting equipment [2, 3, 10].

Pharmacies are an important source of sterile needles and syringes for PWID throughout the world. Available evidence from other countries suggests that pharmacy-based NSP (PBNSP) can be effective in reducing HIV transmission among PWID [11–13]. In Tajikistan, existing regulations do not directly prohibit sales of syringes without a prescription, and numerous pharmacies can be easily accessed in all cities and towns. Therefore, PWID may buy syringes in pharmacies if NSP services are unavailable in the area or if injection equipment is needed when NSPs are closed. Nevertheless, PWID reported cases of pharmacists refusing to sell syringes to PWID, mistreating them, or reporting them to the police [10]. Difficulty in accessing syringes in pharmacies is further compounded by vague or unfavourable policy frameworks. In particular, since governmental policies do not explicitly allow the provision of syringes without a prescription for prevention purposes, legislation prohibiting aiding and abetting drug use may be used to prosecute service providers who offer syringes to PWID [14, 15].

Publications from other parts of the world suggest that pharmacists’ stigmatization of PWID plays a critical role in limiting access to sterile injection equipment and other services [16–21]. Stigma is a complex phenomenon. In their seminal work ‘Conceptualizing Stigma’, Link and Phelan defined stigma as a process involving ‘…co-occurrence of [stigma] components – labeling, stereotyping, separation, status loss, and discrimination’ and emphasized that ‘…for stigmatization to occur, power must be exercised’ [22], p. 363. According to the authors, the process of stigmatization starts with labelling salient human differences. They highlight that the term ‘labelling’, in contrast to ‘mark’, ‘condition’, or ‘attribute’, indicates that labels are assigned as the product of social processes and are not a valid designation of a stigmatized persons’ characteristic. The label ‘drug addict’ implies that addiction is a key characteristic of a person that overshadows his or her other traits. Further, labelled persons are associated with undesired characteristics according to the prevailing cultural norms and beliefs (negative stereotyping). Stigmatization then entails separation of labelled persons from the rest of society by placing them into a different category (‘us vs. them’) and finally loss of status and discrimination, which is an integral part of stigma. In 2004, Link and colleagues added the emotional reactions of stigmatizing and stigmatized persons as one more component of stigmatization [23].

Stigma does not develop in a vacuum but closely depends on the sociocultural context of the society; stigma exists as part of social relationships, as indicated by Goffman [24]. Drug-related stigma research conducted in other parts of the world may have limited applicability in understudied areas such as Tajikistan that feature a unique sociocultural and historical context. Examples of contextual factors influencing drug-related stigma include legacy of Soviet-era prohibitionist drug policies, collectivistic and patriarchal norms, and the rising influence of Islam [25–28]. Islam prohibits the use of mind-altering substances, so it may be the most important cultural factor defining stigma against PWID and thus hindering prevention programmes in this predominantly Muslim region, as was found in other predominantly Muslim countries [29]. Furthermore, the role of Islam in shaping attitudes towards PWID may be particularly salient in the patriarchal collectivistic societies of Central Asia that have little tolerance for deviations from social norms [30]. Therefore, investigating factors underlying pharmacists’ attitudes towards PWID in Tajikistan may be an important step forward in developing effective anti-stigma interventions locally. Furthermore, rapid changes in the sociocultural context in transitioning countries, such as changing norms or exposure to new information, may result in generational differences in stigma perception [31], thus warranting exploration of PWID-related stigma among both current pharmacists and pharmacy students.

To address these research needs, our qualitative study aimed to explore the meanings and processes of stigmatization and discrimination of PWID by pharmacists and pharmacy students in Tajikistan via the prism of the local sociocultural context to inform promotion of prevention services for this population.

## Methods

### Setting

We conducted individual in-depth interviews with pharmacists and pharmacy students in the cities of Dushanbe and Kulob in Tajikistan in November–December 2014. Dushanbe, the capital city with the population of 776,000 (the largest in the country [32]), was selected as a study site because it contains the largest PWID population in the country [3]. Kulob (population 100,000 [32]) was chosen because it is the largest city in the Khatlon province that borders Afghanistan. Both cities have a wide range of free HIV-related services targeting PWID including NSP, pilot opioid substitution treatment (OST) programmes, and HIV testing and treatment [2].

### Participants

Eligibility criteria for participants were living in Dushanbe or Kulob, being age 18 or above, able to provide informed consent, and either working as a pharmacist in a pharmacy or being a student in a university pharmacy department. We aimed to recruit 30–35 participants. Pharmacists were recruited in pharmacies; purposive sampling was employed to achieve diversity in location of pharmacies within each city and to achieve diversity in the age and gender of pharmacists. Pharmacy students were recruited via informational fliers distributed at the Pharmacy Department of Tajik State Medical University located in Dushanbe. Eligible candidates provided informed consent and received cash compensation equivalent to US$10. The Emory University IRB and Medical Ethics Committee of the Ministry of Health and Social Protection of Tajikistan approved the study protocol.

### Data collection

The first author (UI) conducted interviews in Tajik and Russian using a semi-structured interview guide. The guide covered personal attitudes towards drug use and PWID, encounters with PWID, awareness and beliefs related to drug dependence and HIV, and attitudes and practices related to providing syringes to PWID. The guide’s topics were reviewed and revised iteratively between interviews. We stopped conducting interviews when thematic saturation for stigmatization of PWID had been reached. Interviews with pharmacists took place in pharmacies (either in a back office or after the pharmacy had closed), while the students were interviewed in a room in the Pharmacy Department of the Tajik Medical University. All interviews were audio-recorded with participants’ consent.

### Analysis

Audiotapes of interviews were transcribed verbatim into Tajik and Russian. Transcripts were analysed using theoretical thematic analysis methods, a method that codes data and groups codes into themes using a predefined theoretical framework [33]. In particular, we mapped emic codes (i.e. codes identified and labelled based on data, not theory) derived from the data into theoretically defined themes related to the stages of the stigmatization process (stereotyping, emotional reaction, labelling, loss of status, isolation, and discrimination) proposed by Link, Phelan, and colleagues [22, 23]. In addition, we created a separate theme for underlying norms and beliefs related to participants’ perception of drug use as stigmatized characteristic.

Two coders fluent in local languages and familiar with the content area coded each transcript using the constant-comparison method, discussing and reconciling coding discrepancies when they arose. Initial emic codes with related meanings were grouped into categories. Finally, more narrowly defined emic categories were grouped into higher level categories corresponding to the processes within Link and Phelan’s stigma concept. We assessed differences in themes and sub-themes between pharmacists and students, as well as across participants’ gender, age, and city. For purposes of this manuscript, the first author translated the quotes presented in the ‘Results’ section to English.

## Results

### Sample characteristics

In-depth interviews were conducted with 19 pharmacists and 9 students (*N* = 28, Table 1). Ten of the pharmacists and all of the students were from Dushanbe; 9 pharmacists from Kulob. The youngest participant was 21; the oldest was 52 (median = 35). The majority of pharmacists (*n* = 12) and students (*n* = 5) were women.

**Table 1:** Demographic characteristics of pharmacists and pharmacy students interviewed in Dushanbe and Kulob cities of Tajikistan (*N* = 28)

Characteristics	*n*	%
Professional status		
Pharmacists	19	67.9
Students	9	32.1
Gender		
Men	11	39.3
Women	17	60.7
City of residence		
Dushanbe	19	67.9
Kulob	9	32.1

### Religion and other underlying beliefs about drug use

Almost all participants, including those who disagreed with using harsh measures towards PWID, viewed drug use as an unacceptable or undesirable behaviour, even when it does not result in harmful consequences. About three quarters of participants referred to Islam’s prohibition against drugs while explaining their negative views of drug use. Moreover, a few participants stressed that by using drugs a person commits a sin, and, therefore, deserves punishment:

[Drugs] destroy the person, destroy his soul. In Islam, a person’s soul should be attached to Allah only. If we use drugs or alcohol, then we would stop thinking about [God]. […] Therefore, a person who uses drugs becomes a sinner. It does not matter why he started using. It matters that he could not stop, could not overcome himself. If we overcome our [weaknesses], then we become righteous [Muslims] in Allah’s eyes. […] We should live by Sharia laws, if we violate them, we become sinners and will be punished [in the afterlife]. - Female student, 22, Dushanbe.

At the same time, more than half of the participants viewed drug dependence as a disease and believed that drug dependence should be treated rather than punished; pharmacists were more likely than students to endorse this view:

I think it is kind of [a] disease, they have this dependence, addiction to drugs… But they are not criminals from the start, they become criminals [by stealing] to find money for drugs… They are like psychiatric patients, if [mentally ill persons] do something, even kill someone, they are not imprisoned, they are sent to a hospital for treatment… I think [PWID] should be treated in the same way too… - Female pharmacist, 34, Dushanbe.

Nevertheless, some of the participants who agreed that drug dependence is a disease stated that it is still a sin and cannot be completely forgiven from an Islamic point of view. Notably, though, a few participants who emphasized their strong belief in Islam stressed that mistreating people who use drugs results from superficial interpretation of Islamic doctrines. According to them, a Muslim’s duty is to support his or her spiritual fellows and avert them from the wrong path. ‘[T]he real Muslims should help their brothers and sisters to overcome this habit’, as a male pharmacist (age 52) from Dushanbe put it. However, a few participants justified ostracizing PWID who do not want to abstain from drugs on religious grounds, since they are ‘persistent in their sin’ (a female student, 22, Dushanbe).

About one third of participants shared that they perceived drug use to be a crime and PWID as criminals; men were more likely than women to have this opinion. They stressed that people who start using drugs are aware of their illegal nature and negative consequences, which, according to these participants, constitutes a criminal behaviour. A few participants justified their position by citing the experiences of other countries that imprison or even execute PWID. Overall, participants who held negative perceptions of drug use and dependence often were in favour of harsh treatment of PWID, as discussed below.

### Labelling of PWID

Participants provided examples of labels affixed to PWID by society—*narcoman* (a medical term denoting a person dependent on drugs introduced by Soviet medicine), *nash’amand* (drug addict in Tajik), and *gershik* (Russian slang name for heroin user). All participants mentioned that these names have strong negative connotations and are closely linked to negative stereotypes of PWID. A few participants mentioned that *narcoman* means a person with no purpose in life other than using drugs. All participants shared that attaching labels of *narcoman* or *gershik* to a person results in lowering his or her status in the society. In addition to labels directly related to drug use, participants attached labels denoting the deviation of PWID from religious, moral, and social norms (e.g. *sinners*, *life destroyers*, *criminals*, *madmen*).

### Stereotyping PWID

#### Crime, aggression, and violence

One of the most frequently cited stereotypes of PWID was that they have a high propensity for crime and violence. While almost all participants mentioned that PWID may commit violence to find money for drugs, about a third of participants also shared that PWID may inflict harm to innocent people for no rational reason. These participants were also more likely to support punishment and institutionalization of drug users in mandatory treatment facilities. Some of these participants, mostly pharmacy students, believed that it is a direct effect of drugs that makes an individual violent: ‘After they inject, they become insane and can do anything. They can kill or rape someone’ (a male student, 21, Dushanbe). A few participants also believed that PWID may force others into drug use just to increase the number of PWID. One of these participants was particularly vivid in portraying PWID as irrationally aggressive individuals:

Most of them are night-walkers, these drug users, so they may kidnap people who go out at night and forcefully inject them [with drugs], maybe to expand the circle of drug users. [PWID will inject] forcefully, with used syringes, sending the drug straight into their vein. - Female student, 21, Dushanbe.

Notably, such exaggerated stereotypes of PWID were reported mostly by the students rather than by pharmacists. While most of the participants did not share such extreme views of PWID, many still noted that PWID are prone to involve others in drug use to profit from selling drugs to them.

#### Harm to family

All participants believed that PWID harm their families; almost half of them emphasized this as one of the main reasons for their negative attitudes towards PWID. According to the participants, PWID neglect parents, wives, and children; steal from home or sell household belongings; trigger quarrels and commit violence against family members; and drain family financial resources. In addition, PWID affect the reputation of their families, and their relatives may suffer from stigma by association.

Almost all participants noted that PWID are bad parents. According to them, PWID fail to support their children financially, and create an adverse emotional environment by causing quarrels and violence. A few participants also mentioned that PWID may put the health of their children at risk via mother-to-child transmission of blood-borne infections or via accidental needle-stick injuries from used syringes. A few participants suggested depriving PWID of parental rights, at least till they stop using drugs.

…[PWID] cannot raise good children. Their kids are always wandering on the streets, they also get used to stealing, fighting, cursing, lying since childhood, then they become criminals. If [a PWID] has some good relatives, [children] should be given to them. If not, kids should be taken to an orphanage, until their parents stop [using drugs]. Or, say, if a husband uses [drugs], then his wife should divorce him and take away the kids with her. - Female pharmacist, 46, Dushanbe.

#### Harm to society

Several male pharmacists and students and one female student justified their negative attitudes towards PWID by the damage they cause to the society and the country. According to them, PWID harm society by involving others in drug use, stealing and committing other crimes, not working productively, and affecting the future of the nation by bearing sick children. Participants who held such views were more likely to support harsh measures against PWID.

[PWID] ruin our society. When foreigners come to Tajikistan, they see [high rate of] drug use, they laugh at us, say we are a backward country […]. This affects the reputation of our country. So let them be forced [to treatment], especially those [PWID] who lay on the streets […]. - Male student, 23, Dushanbe.

Notably, a few participants stressed that not all PWID are inherently bad and acknowledged that there may be good people using drugs. Nevertheless, these participants also believed that even being a good person, PWID may harm other people.

#### Controllability of drug use

About two thirds of participants who discussed the role of willpower in drug abstinence believed that an addicted person could stop using drugs if he or she truly wants to. However, most of these participants reported that PWID are weak, unmotivated people. These participants were also more likely to hold the view that drug use is a crime and a sin that deserves punishment:

The best solution to the problem is that PWID should stop using drugs. They should summon their willpower, should overcome their weakness, start managing their lives. But most of them are just weak people who want to indulge themselves. - Male pharmacist, 27, Dushanbe.

On the other hand, about one third of participants (most of whom also believed that drug use is a disease), pointed out that stopping drug use only partially depends on the willpower of PWID; this view was particularly common among older pharmacists. They listed other factors such as peer pressure, support by family members, access to treatment, education, and employment that may ensure sustainable abstinence.

### Emotional responses

One of the common emotions triggered by PWID in participants was fear. Almost all of the participants who were asked to compare their perceptions of PWID and alcoholics indicated that they were more afraid of PWID. Participants mentioned that people may be afraid of crimes committed by PWIDs to acquire money to buy drugs or fear PWID involving others into drug use. Several participants (mostly women) were more fearful of PWID who may initiate participants’ children into drug use:

God forbid if some of these drug users would approach my sons, would give them drugs, would deceive them. I am always worried about it when I hear that a young guy started [using drugs]. Any parent would be afraid of it. - Female pharmacist, 49, Kulob.

Almost two thirds of participants also mentioned aggressive behaviour of PWID when they are under the influence of drugs or in withdrawal, as the reason they fear PWID. A few mentioned disgust and contempt towards PWID.

On the other hand, about one third of men and more than half of women in the study reported feelings of pity and sympathy towards PWID because they ruin their young lives, commit sin by using drugs, harm their health, deprive themselves of positive life experiences, and die early. Participants who had relatives or friends who injected drugs tended to feel sympathy or pity towards them, while participants who encountered PWID only at pharmacies talked mostly about fear and disgust.

### Status loss and discrimination

#### Rejection and ostracism

All participants reported that PWID are ostracized by their families, friends, and neighbours. People try to avoid PWID, stop inviting them to community events, or show them less respect (e.g. by seating them closer to doors in the room where a party or a meeting is taking place). Sometimes parents may publicly renounce their children if they find them using drugs. Families of PWID may also be rejected by community members. In particular, since arranged marriages are common in Tajikistan, young men and especially women from PWID families may have bleaker marriage prospects because of stigma:

[People’s] attitude towards families [of PWID] may get worse as well. People will have less [contact] with them. Nobody would take a girl from their family [in an arranged marriage] or give their daughter to [a family of a PWID], people would see them as tainted. […] Everyone will be gossiping about them.

- Female pharmacist, 34, Dushanbe

Several participants who believed that drug use is a sin against Islam indicated that PWID should not be allowed to visit mosques, since ‘their prayers cannot be accepted when they use drugs’ (male pharmacist, 45, Kulob). However, almost all participants who were asked this question disagreed with this position. Several participants were of the opinion that ‘in the mosque, [a PWID] can pray, listen to preaching and persuasion of others, this can help him to stop [using drugs]’ (female student, 22, Dushanbe).

Although about three quarters of participants supported harm reduction programmes, with pharmacists more likely to support these programmes than students, in general, some of them shared that they are against spending public resources on PWID who do not want to stop using drugs. Rather they would allocate available funds to primary prevention of drug use:

If they still keep using, then why waste all that money on them? If I had all that money [at my disposal], I would open more sports facilities, so kids do not do anything [illegal], so they do not hang out somewhere smoking [marijuana]. I would rather spend money for [prevention], than support chronic addicts who keep on using.

- Male student, 22, Dushanbe.

Many participants viewed PWID, in particular those who do not want to stop using drugs, as individuals with worthless lives and no future. A few participants, mostly men, stressed that death of PWID from overdose or other reasons is the best outcome for everyone. At the same time, the vast majority of participants (including a couple of those who believed that death is the best outcome for PWID), when asked, stressed that each life, including that of a PWID, must be valued and mentioned that wishing death to someone is against Islam.

#### Isolation

About one quarter of participants (mostly men) indicated that PWID should be isolated from the rest of society via imprisonment or forced treatment. They reasoned that isolation would prevent PWID from committing crimes, involving others in drug use and transmitting blood-borne infections. A few of them added that isolation would be beneficial for PWID themselves as it would sever their ties with drug dealers and thus would help PWID to recover from addiction. Most of the participants who believed that drug use is a crime were supportive of forced treatment, isolation and even imprisonment of PWID, if the latter do not want to receive treatment and stop using drugs. One participant referred to the experience of Soviet system of treatment and labour facilities (LTP - transliteration of the Russian acronym for ‘treatment and labour facilities’) run by police:

[PWID] are sick, so they should be treated. But if they do not want to [be treated], or want to continue using drugs, then police should force them [to get treated]. [Government] should establish something like LTP, you know from old times, right? Like a prison, but not a prison, so they get their medicines strictly by regimen and no contacts [with outside] without permission. Let them work there after [an acute withdrawal] passes. Not a very hard work, nor an easy one, he should sweat a bit there. You will see, in a year he will get well there, he will not have cravings [for drugs] anymore. If he starts earning [for his work] there, his life will improve, he will [reconcile] with his family, he definitely will turn away from that path. - Male pharmacist, 47, Dushanbe.

On the other hand, majority of participants disagreed with isolation and forced treatment of PWID. Most of them mentioned the ineffectiveness of isolation, pointing out the inability of treatment providers to ensure complete isolation and the need to address underlying problems, such as unemployment or psychological problems that resulted in drug addiction. A few participants stressed that isolation is inhumane and should not be applied to PWID.

### Power and stigma

Almost all participants who were asked about the role of income in stigmatizing PWID shared that richer PWID experience less stigma from community members compared to poorer ones. They reasoned that more affluent PWID do not need to steal, borrow, or beg for money to purchase drugs. In addition, participants mentioned that affluent people can help others and in general have higher status in the communities which also lessens stigma towards them:

The rich guy [who uses drugs is not stigmatized], but the poor guy definitely is. The rich guy, he has money, he does not depend on anyone, he manages his livelihood, he may even help his neighbours [financially]. Even if they know about [his using drugs], they will not tell him anything, they will show him respect, but maybe they will talk behind his back. But if a poor guy [uses drugs], he has no money, so he has to do some [inappropriate] things, like stealing, or, say, selling family belongings, or borrowing money. So, he will lose respect in the eyes of others, they can say bad things into his face.

- Male pharmacist, 33, Dushanbe.

At the same time, participants who reported that attitudes of community members depend on PWID income level, stressed that they personally treat rich and poor PWID in the same way.

### Societal stigma

All participants indicated that society in general has very negative attitudes towards PWID. Those participants who reported positive or neutral attitudes towards PWID mentioned that other pharmacists as well as other society members treat PWID significantly worse than they did:

Sometimes I see, say, in other pharmacies, [a PWID] comes and asks for a syringe. And the pharmacist says, ‘Go away, I don’t have any [syringes]’. They talk to [PWID] in a very rude way, tell them to get lost. Well, [a PWID] is still a human, even if he does [drugs], you should support him. - Male pharmacist, 32, Kulob.

Almost half of the participants believed that the stigmatization of PWID further isolates them and hampers treatment and recovery, and some participants were against such maltreatment of PWID; these participants also were more likely to believe that drug dependence is a disease. However, several participants opined that the stigmatization of PWID plays a positive role as a drug use prevention strategy, since it sends a clear message to others to avoid drug use.

Well, bad treatment of [PWID] is a good thing, it lets others see it and learn a lesson… Because, if [attitudes] get positive, then people will not be afraid [of using drugs]. […] If children see that people treat [PWID] badly, then they will think, ‘Aha, nobody likes drug users, so let’s stay away from [drugs]’. Otherwise, he would say, ‘Well, looks like it is not a big deal, I can try’. […] Of course, [stigma] makes life harder for drug users, but on the other hand, I think it is more important not to let others to start [using drugs].

- Male pharmacist, 52, Dushanbe.

### Access to syringes

While fewer than half of pharmacists and students acknowledged encountering PWID outside of pharmacies, almost all pharmacists had experience interacting with PWID in the pharmacies. A customer is suspected to be PWID when he or she asks for a syringe and an ampule of Dimedrole (brand name of antihistamine Dyphenhidramine) or Novocaine (local anaesthetic). These two medicines can potentiate the effect of heroin and are used to dissolve heroin. A few participants also mentioned that PWID can be recognized by their wary or agitated gaze or a specific body odour, though the participants could not describe the smell.

Many male participants and some women, most of them from Kulob, stressed that they do not or would not sell syringes if they know or suspect the customer uses drugs. Often the reasons for refusing syringes were related to participants’ negative perceptions of drug use and/or people who use drugs. Almost all of these participants saw providing syringes to PWID as encouraging drug use. Moral responsibility for encouraging drug use was a recurring theme. Many participants mentioned that they understood the benefits of providing syringes but preferred someone else to do it to avoid the moral burden:

You have to be morally prepared to sell syringes to a drug user… A person should be morally strong (laughter), [reasoning] like, ‘OK, I will sell the syringes, the main thing is to prevent transmission [of HIV]’. But I cannot do it… If you think rationally, [providing syringes] may prevent HIV, this is an advantage. […] But […] if you realize that you sell the syringe and [a PWID] injects with it, it means you helped him [to use drugs]. So it is easier to think that it was not you who [sold the syringe], let it be someone else. Let [NSP] operate, but it is not you who sells the syringe, but someone else, because it is morally hard… -Female student, 23, Dushanbe.

Since many PWID die from overdose, a few participants considered drug use to be a suicidal action and stressed that they did not want to be associated with it, in particular since Islam condemns suicides. More than a few pharmacists also mentioned that they did not sell syringes to PWID because of damage the former may inflict on the pharmacy and disrupt business by improper conduct, stealing and aggression towards pharmacists or customers. Several pharmacists, in particular those from Dushanbe, mentioned that state agencies (Pharmacy Monitoring Service, counter-narcotic law enforcement) may send their informants (some of whom may be PWID) to buy syringes from pharmacists and then impose sanctions for selling syringes without prescription. However, only one pharmacist from Dushanbe acknowledged that he personally had experienced such a situation.

A few participants, mostly those who felt sympathy towards PWID, also noted that they did not sell syringes to PWID acting in PWID’s best interests. Limited access to syringes may help PWID stop using drugs or avoid overdose, explained they. Another set of reasons for not selling syringes to PWID was related to syringe sale policies and enforcement of these policies. A few pharmacists believed strongly that current policies prohibit syringe sales without prescription while several others believed that regulations explicitly prohibit sale of syringes to PWID.

Participants also shared reasons for selling syringes. A few pharmacists stated that they sold syringes out of sympathy to PWID who may otherwise suffer from not having a syringe to inject. One of these participants also added that PWID did not choose to be addicted and cannot control their habit.

In general, pharmacists were more likely to provide pragmatic explanations for their decisions related to selling syringes, while students more often referred to moral or emotional reasons. Pharmacists who reported selling syringes without prescriptions mentioned that selling syringes is part of their business, or that refusing to sell syringes would not prevent PWID from buying them in another pharmacy. At the same time, students who supported provision of syringes to PWID mentioned HIV prevention as well as sympathizing with dire conditions of PWID in withdrawal. Pharmacists who were against selling syringes to PWID often cited potential problems with law enforcement or with PWID disrupting pharmacy operations. Conversely, students tended to explain their reluctance to sell syringes by moral burden related to encouragement of drug use.

Overall, few participants were completely supportive of or totally against providing any access to syringes. About three fourths of participants agreed that sterile syringes should be provided in one way or another to PWID. Most of them supported the idea of distributing syringes via designated community-based NSPs, stressing that these services are endorsed by the government as HIV prevention programmes. However, the majority of these participants were against running NSPs in their pharmacies. Specifically, most of them mentioned that free distribution of syringes would cause an influx of PWID to the pharmacy resulting in theft, debauchery, or disruption of business. Almost all participants pointed out that state bureaucracy and frequent audits are an impediment for pharmacy-based syringe distribution; more than a few also mentioned high workload and lack of training to deal with PWID. A few women pharmacists cited fear of PWID and husbands’ objections as reasons against working in pharmacy-based NSPs.

## Discussion

Our study is the first to explore stigmatizing attitudes of pharmacists and pharmacy students specifically and service providers in general towards PWID in Central Asia. We identified several sub-themes related to stigma and discrimination against PWID by pharmacists and pharmacy students in Dushanbe and Kulob, Tajikistan, that are aligned with Link and Phelan’s conceptualization of stigma [22, 23]. We found that pharmacists and students assign PWID labels with negative connotations, hold negative stereotypes, and express negative emotions to PWID. Our participants brought examples of stigma enacted by themselves or other individuals, including rejection and isolation of PWID, supporting forced treatment, and refusing access to syringes and other resources and services.

Our study suggests that stigma plays an important role in pharmacists’ refusal to provide syringes to PWID. Stigma processes may interact with pharmacists’ decision-making via various pathways. For instance, labelling PWID as sinners leads to perception of selling syringes to PWID as abetting sinful activities. Fear of PWID triggered by negative stereotypes about them as individuals prone to crime, aggression and inflicting damage also reduces willingness to interact with PWID, including selling syringes. By holding stereotypes of PWID as individuals who choose to destroy their lives, families, and society, pharmacists see selling syringes to them as encouragement of these moral transgressions. Similar findings were described for pharmacists throughout the world, though these studies were conducted outside of Central Asia [16–21].

In line with Link and Phelan’s concept, power, and in particular affluence of PWID, may play a moderating role in expressing and enacting stigma towards PWID, with rich individuals eliciting less negative reactions and enjoying higher social status. Another example of the power differential that enables discrimination is the power of pharmacists to decide whether to sell syringes to a person who may ‘look’ or ‘act’ like a PWID (e.g. customers with anxious facial expression, or those who ask for a syringe and injectable antihistamine) or not.

From a theoretical standpoint, our findings suggest that the conceptual model of stigma proposed by Link and Phelan is applicable to drug use in the context of Tajikistan. At the same time, while stigma is acknowledged as a complex sociocultural phenomenon, there is a paucity of studies analysing the impact of the sociocultural context on drug use stigma in developing Muslim countries such as Tajikistan. Therefore, it is important to discern broader sociocultural contextual factors explaining why and how the local context enables and maintains stigmatization of PWID in this country.

Collectivism, or the tendency of individuals to view themselves as part of the whole (e.g. family, society), is one such contextual factor that may contribute to the particular nature of stigma against PWID in Tajikistan. Due to tight interdependence and high levels of surveillance among their members, collectivistic cultures harbour strictly defined and strongly enforced social norms and group values. Therefore, the range of acceptable lifestyle choices in such cultures is narrower and behaviours deviating from traditional norms are less tolerated [34–36]. Empirical studies in China and the UK have shown associations between collectivist cultural norms and HIV and mental health stigmas [37, 38] that are symbolically close to drug use stigma [39, 40]. Similar to other developing countries in Asia and Africa, society in Tajikistan is collectivistic and patriarchal, with group values and norms prevailing over individual choices and opinions [28]. Our participants’ disapproval of drug use even when no direct harm is inflicted on anyone may stem from such a collectivistic mindset, which might consider abstinence as the best outcome. Similarly, the stereotyped views of PWID as purposeless individuals ruining their own lives may be fuelled by collectivistic resentment of the perceived disregard of common values of well-being and prosperity. Specifically, in the eyes of collectivistic society individuals who presumably harm themselves with drug use also inflict damage to collectives (families and society) to which they belong.

Our study suggests that religion is yet another sociocultural factor influencing Tajikistani pharmacists’ attitudes towards PWID. Islam’s prohibition of drug use has triggered strong opposition to harm reduction programmes in some Islamic countries [29]. Some of our participants, too, viewed drug use as a sin and believed that providing syringes to PWID is abetting a sin. However, it would be precipitous to conclude that providers’ religiosity presents an insurmountable barrier for providing harm reduction services. We found that service providers with a high level of religiosity may perceive helping fellow Muslims who inject drugs as their spiritual duty. These findings imply that religious teaching can be applied to advocate for harm reduction services. Positive examples can be found in Malaysia and Iran, where harm reduction programmes referred to Islamic values such as the prescription to protect the ‘faith, life, intellect, progeny and wealth’ of every Muslim [41].

Our findings show that collectivism, paternalism, and religion, key elements of Tajikistan’s sociocultural context, may contribute to the emergence and manifestation of stigma towards PWID in this country. Since adherence to social norms is at the core of each of these sociocultural constructs, it is likely that they do not influence stigma independently from each other but are mediators of a more distal and overarching psychosocial phenomenon. Social and moral conservatism that encourages and enforces adherence to existing social and moral norms can fuel stigma against PWID in Tajikistan and other countries with similar sociocultural environments. Literature shows that social conservatism is positively associated with vertical collectivism and religiosity, and that all these factors are correlated with stigma and prejudice [42–46].

Further, conservative worldviews can also be linked to stigmatizing PWID through the stereotype of the controllability of drug use [47], a sub-theme that emerged in our study as well. We found that a majority of our participants believe PWID are personally responsible for their drug using habits and lack the willpower to abstain. At the same time, these beliefs suggest that pharmacists and pharmacy students may have limited knowledge about the nature of drug dependence and that further training is required to address myths and misconceptions about drug dependence.

The influence of the cultural context, including socially conservative and collectivistic norms, on stigmatization of PWID may be moderated by individual characteristics. Thus, differences in stereotypes related to PWID or rationales behind the decision to sell or refuse syringes may be explained by differences in students’ and pharmacists’ life and professional experiences, including various level of exposure to PWID. Generational differences in expressing stigma against marginalized populations at risk for HIV were found by Balabanova et al. [31] for Russia.

In addition to the cultural context, we also identified how policy-related structural factors shape stigma and discrimination against PWID in Tajikistan. Ambiguous policies regulating syringe sales allow pharmacists to arbitrarily refuse to sell injecting equipment to PWID. Another example is use of the derogatory label *narcoman* for PWID. It should be noted that *narcomania* was an official diagnosis assigned by Soviet medicine to people with drug dependency [48]. *Narcomania* is still the official term for drug dependence used in Tajikistan’s *Law on Narcological Aid*, adopted in 2003. Use of derogatory labels by well-meaning service providers may indicate a lack of locally accepted neutral terms to denote people with drug dependency problems and highlights the need for introducing more neutral terms into public health policies and practice.

Our findings highlight the importance of background contextual factors such as culture and policies in analysing sources of stigma and its impact on the health of marginalized populations. Our study is one of the initial steps to fulfil this gap for Tajikistan, and, more broadly, for the region of post-Soviet Central Asia. Further research should use quantitative designs to assess the relationships between sociocultural context, policies, stigmatization of PWID and their access to prevention services. Public health programmes should undertake a multipronged approach to promoting the accessibility of sterile injection equipment. These activities include teaching pharmacists and students about harm reduction principles; revising policies regulating syringe sales and legislation on aiding and abetting drug use so that they clearly allow sales, and ensuring that all relevant parties are aware of their stipulations; and disseminating culturally appropriate messages addressing the faith-based concerns of pharmacists and other service providers. Educational and advocacy activities should involve and be delivered in collaboration with the National Pharmacy Monitoring service, law enforcement, and religious leaders.

### Strengths and limitations

We assessed the study strengths and limitations utilizing Maxwell’s [49] framework of qualitative research validity. To enhance the study findings’ descriptive validity, all interviews were audio-recorded, transcribed verbatim and analysed in the original languages. The first author (UI), a native of Tajikistan deeply familiar with the local socio-cultural context, conducted all interviews and analysed the results, thus enhancing interpretative validity. However, the theoretical validity of the study results may be limited since categorization of codes into predefined stigma constructs might have not taken into account subtle variations in processes and meanings related to participants’ attitudes towards PWID. In addition, the number of student participants was not large enough to reach a theoretical saturation of stigma-related themes among this subsample.

## Conclusions

Our study is the first to explore stigmatization of PWID by service providers in post-Soviet Central Asia. We demonstrated the multifaceted nature of stigma against PWID in Tajikistan and its role in shaping pharmacists’ attitudes towards provision of services to this population. Our findings suggest that the local sociocultural context, in particular religious beliefs, collectivistic mentality and, in a broader sense, social conservatism, may facilitate stigmatizing beliefs and attitudes. We have also shown how structural factors, such as policies and their implementation, may tip the power balance between pharmacists and PWID and affect the accessibility of syringes for PWID. Although the global literature offers conceptual models outlining the effect of contextual and structural factors on stigma, there is a lack of empirical evidence supporting applying these models to developing countries and understudied cultures. Our study is the first step in addressing this gap for the countries of post-Soviet Central Asia.

## Abbreviations

HCV: Hepatitis C virus
HIV: Human immunodeficiency virus
NSP: Needle and syringe programme
OST: Opioid substitution therapy
PBNSP: Pharmacy-based needle and syringe programme
PWID: People who inject drugs
